# Effects of powdered Montmorency tart cherry supplementation on acute endurance exercise performance in aerobically trained individuals

**DOI:** 10.1186/s12970-016-0133-z

**Published:** 2016-05-26

**Authors:** Kyle Levers, Ryan Dalton, Elfego Galvan, Abigail O’Connor, Chelsea Goodenough, Sunday Simbo, Susanne U. Mertens-Talcott, Christopher Rasmussen, Mike Greenwood, Steven Riechman, Stephen Crouse, Richard B. Kreider

**Affiliations:** Department of Health and Kinesiology, Exercise and Sport Nutrition Laboratory, Texas A&M University, College Station, TX 77843-4243 USA; Department of Nutrition and Food Science, Institute for Obesity Research and Program Evaluation, Texas A&M University, College Station, TX 77843-4243 USA; Department of Health and Kinesiology, Human Countermeasures Laboratory, Texas A&M University, College Station, TX 77843-4243 USA; Department of Health and Kinesiology, Applied Exercise Science Laboratory, Texas A&M University, College Station, TX 77843-4243 USA

**Keywords:** Recovery, Antioxidants, Anti-inflammatory, Muscle damage

## Abstract

**Background:**

The purpose of this study was to determine whether short-term supplementation of a powdered tart cherry supplement prior to and following stressful endurance exercise would affect markers of muscle damage, inflammation, oxidative stress, and/or muscle soreness.

**Methods:**

27 endurance-trained runners or triathlete (21.8 ± 3.9 years, 15.0 ± 6.0 % body fat, 67.4 ± 11.8 kg) men (*n* = 18) and women (*n* = 9) were matched based on average reported race pace, age, body mass, and fat free mass. Subjects were randomly assigned to ingest, in a double-blind manner, capsules containing 480 mg of a rice flour placebo (P, *n* = 16) or powdered tart cherries [CherryPURE®] (TC, *n* = 11). Subjects supplemented one time daily (480 mg/day) for 10-d, including race day, up to 48-hr post-run. Subjects completed a half-marathon run (21.1 km) under 2-hr (111.98 ± 11.9 min). Fasting blood samples and quadriceps muscle soreness ratings using an algometer with a graphic pain rating scale were taken pre-run, 60-min, 24 and 48-h post-run and analyzed by MANOVA with repeated measures.

**Results:**

Subjects in the TC group averaged 13 % faster half-marathon race finish times (*p* = 0.001) and tended to have smaller deviations from predicted race pace (*p* = 0.091) compared to P. Attenuations in TC muscle catabolic markers were reported over time for creatinine (*p* = 0.047), urea/blood urea nitrogen (*p* = 0.048), total protein (*p* = 0.081), and cortisol (*p* = 0.016) compared to P. Despite lower antioxidant activity pre-run in TC compared to P, changes from pre-run levels revealed a linear increase in antioxidant activity at 24 and 48-h of recovery in TC that was statistically different (16–39 %) from P and pre-run levels. Inflammatory markers were 47 % lower in TC compared to P over time (*p* = 0.053) coupled with a significant difference between groups (*p* = 0.017). Soreness perception between the groups was different over time in the medial quadriceps (*p* = 0.035) with 34 % lower pre-run soreness in TC compared to P. Over the 48-h recovery period, P changes in medial quadriceps soreness from pre-run measures were smaller compared to TC.

**Conclusion:**

Results revealed that short-term supplementation of Montmorency powdered tart cherries surrounding an endurance challenge attenuated markers of muscle catabolism, reduced immune and inflammatory stress, better maintained redox balance, and increased performance in aerobically trained individuals.

## Background

Acute bouts of strenuous aerobic exercise facilitate a stress response characterized by mechanical muscle damage, oxidative stress, and inflammation that parallels the physiological stress response associated with many adverse traumatic cardiovascular events and illnesses [1–3]. As a result, this type of long duration mechanical muscle stress and high oxidative metabolic demand [4], significantly increases free radical production beyond the capacity of the endogenous antioxidant systems. Ultimately, this increase facilitates excessive cell damage, altered cell signaling [5–7], decreased cellular performance [5–8], lipid peroxidation, oxidation of proteins and glutathione, and subsequent DNA damage [3, 9]. Exercise-induced muscle soreness is indirectly related to inflammation as a product of high nociceptor and mechanoreceptor sensitivity to potent metabolites released during muscular degeneration [10, 11].

The use of antioxidant supplements, such as vitamins C [12–15] and E [4, 14, 15], in athletic applications to help fortify the body’s endogenous antioxidant response has spurred some success. However, vitamins C and E (independently or in combination with N-acetylcysteine, β-carotene, or α-lipoic acid) remain controversial due to conflicting reports of effectiveness [3, 16–19] with potential post-exercise pro-oxidant effects on muscle protein anabolism [20–22], endogenous antioxidant capacity [22], and mitochondrial biogenesis [23].

More recent nutritional research has focused on the antioxidant effects of functional foods containing high concentrations of phenolic compounds such as flavonoids and anthocyanins. It is proposed that these may act synergistically with other compounds contained within the food to provide an overall aerobic exercise recovery benefit [4, 24]. A wide variety of antioxidant and polyphenol-containing functional foods such as grape extract [25], chokeberries [26], and blueberries [8] have shown performance-enhancing and exercise recovery benefits. Exercise-based research with similar functional foods spurred investigation with tart (e.g. Mortmorency) cherry concentrate and juice supplementation to help increase performance by theoretically attenuating muscle damage, oxidative stress, and inflammation associated with aerobic challenges [7].

There are a few studies that have evaluated the effects of tart cherry supplementation on responses to endurance-based exercise. The first endurance-based study investigated the effects of 8-d tart cherry cultivar-blended juice supplementation on exercise-induced muscle pain surrounding an endurance relay race event (running distance = 22.5–31.4 km) [27]. Exercise-induced muscle pain was reduced as a result of tart cherry supplementation, but the findings were not confirmed by subsequent blood marker analysis [27]. Following a similar 8-d tart cherry juice supplementation protocol, a second study reported greater lower body isometric strength and quicker restoration of muscular function with reduced blood markers of muscle damage, oxidative stress, and inflammation in response to a marathon run [28]. A third endurance study examined the effects of 7-d tart cherry concentrate supplementation on physiological markers of muscle damage, oxidative stress, and inflammation surrounding 3-d of simulated high-intensity road cycling [4]. Similar to the second study, reductions of oxidative and inflammatory responses were the primary findings, thereby demonstrating a potential acute recovery-enhancing effect between bouts of high-intensity aerobic exercise with tart cherry supplementation [4].

The primary objective of this study was to determine whether short-term (10-d) supplementation with a powdered form of tart cherry skins would facilitate greater aerobic performance through attenuation of oxidative stress, inflammation, muscle damage, and muscle soreness.

## Methods

### Subjects

**Fig. 1:**
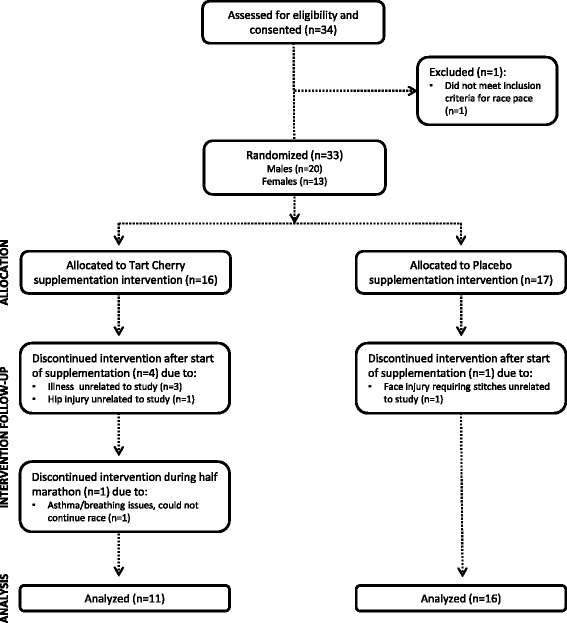
Consort diagram breakdown of the subject population from recruitment to data analysis

Twenty-seven male (*n* = 18) and female (*n* = 9) endurance-trained runners or triathletes (21.8 ± 3.9 years, 67.4 ± 11.8 kg, 15.0 ± 6.0 % body fat, 51.2 ± 11.4 kg free fat mass) participated as subjects in this study. Subjects were recruited through paper and electronically distributed flyers at Texas A&M University. Entrance criteria required the runners or triathletes to have been involved in a consistent running program for at-least 1-year and able to run a half-marathon (21.1 km) in less than 2 h. Figure 1 provides a breakdown of the subject population. Subject discontinuation of participation was not related to any aspect of the supplementation or testing protocol.

All subjects signed informed consent documents and the study was approved by the Texas A&M University Institutional Review Board prior to any data collection. Subjects were not allowed to participate in this study if they reported any of the following: 1) metabolic disorders or taking any thyroid, hyperlipidemic, hypoglycemic, anti-hypertensive, anti-inflammatory (e.g. NSAIDs), and/or androgenic medications; 2) history of hypertension, hepatorenal, musculoskeletal, autoimmune, and/or neurological disease(s); and 3) allergy to cherries or any cherry components (e.g. polyphenols, anthocyanins, anthocyanidins).

### Experimental design

**Fig. 2:**
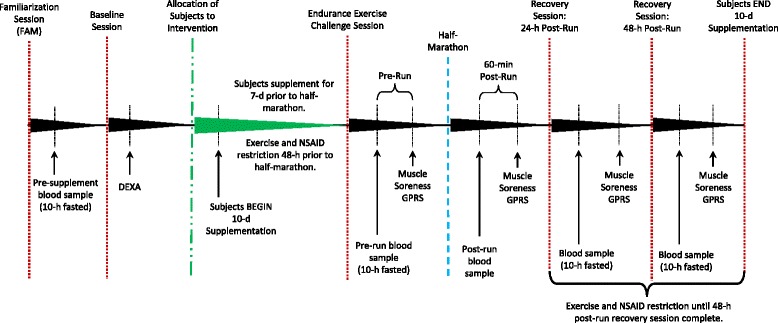
Experimental study design. *DEXA* dual-energy X-Ray absorptiometer, *MVC* maximal voluntary contraction, *1-RM* 1-repetition maximum, *NSAID* non-steroidal anti-inflammatory drugs, *GPRS* graphic pain rating scale, *7-d* 7-day, *48-h* 48-hour

The study was conducted in a randomized, double-blind, and placebo-controlled manner (see Fig. 2). All subjects completed a morning familiarization (FAM) session where they were provided detailed information regarding the study design, testing procedures, and supplementation protocols. Informed consent, medical history, and endurance training history questionnaires were also completed during the FAM session. A nurse reviewed medical history documents and performed a physical exam (resting vital signs and lung auscultation) on each subject to ensure participation eligibility. A fasting blood sample was taken at the end of the FAM session. Approximately 10-d prior to the endurance exercise intervention, subjects returned to the lab for a morning baseline testing session to determine body mass, height, and body composition. Following baseline measurements subjects were matched based on average reported race pace, fat free mass, body mass, and age and randomly separated into two groups: 1) a placebo group or 2) a powdered tart cherry group. Subjects were instructed to not change their dietary habits in any way throughout the study. Nutritional habits were monitored through self-dietary recall for 4-d (3 weekdays and 1 weekend day) of the first seven supplementation days.

Subjects were instructed to begin supplementation 7-d prior to the endurance exercise challenge (Day 0). Subjects were asked to fast overnight for 10-h to account for diurnal variation as well as abstain from exercise and consumption of non-steroidal anti-inflammatory medications (NSAIDs) for 48-h prior to all testing days. On the day of the endurance exercise challenge, the subjects reported to the lab where body mass, resting heart rate, and resting blood pressure were measured. Subjects then donated a fasting venous blood sample (approximately 20 ml) using standard clinical procedures and rated perceptions of muscle soreness to a standardized application of pressure on their dominant thigh at three designed locations using a graphic pain rating scale (GPRS). Twenty minutes prior to the start of the half-marathon race, subjects were allowed to warm-up as they normally would before running a road race. Subjects completed a half-marathon (21.1 km) run outdoors at their normal race/competition pace. Both water and glucose-electrolyte drinks were provided ad libitum to the subjects at regular intervals during the race. Fasting (except 60-min post-run) blood samples and GPRS ratings of quadriceps muscle soreness were completed at 60-min, 24 and 48-h of post-run recovery. The last or tenth day of supplementation correlated with 48-hours post-run recovery.

### Exercise protocol

#### Half-marathon (21.1 km) run

On the morning of supplementation day 8, all subjects performed an outdoor half-marathon run (21.1 km) for best time on a closed course under simulated race day conditions. Race start (0800) conditions were: ambient temperature = 22.8 °C, wind = 14.5 kph, humidity = 90 %, dew point = 21.1 °C. Conditions at the race finish (1030) were: ambient temperature = 25.0 °C, wind = 14.5 kph, humidity = 86 %. The race was run completely on concrete and pavement surfaces. All subjects were given 20-minutes for individual warm-up routines. At regular intervals (4 total locations) throughout the race, fluids (water and/or glucose-electrolyte beverages) were made available ad libitum to the subjects. Each subject had their own water and glucose-electrolyte beverage bottle labeled with a number that corresponded to their race number. All fluid bottles were weighed before and after the race to determine fluid consumption for each subject. Official race splits and finish times were recorded by designated lab staff. Following the race, subjects were not allowed to run to cool down, only stretching and minimal ambulation was permitted until the 60-min post-run testing session.

### Supplementation protocol

Subjects were assigned in a double-blinded and randomized manner to ingest a rice flour placebo (P, *n =* 16) or powdered tart cherry (TC, *n =* 11). Subjects were matched into one of the two groups according to average reported race pace from previous (within the last 1 year) race events, fat free mass, body mass, and age. Subjects were instructed to ingest one 480 mg supplement capsule one time daily directly after breakfast at 0800 for 7-d prior to, the day of, and for 2-days following the half-marathon race for a total supplementation timeline of 10-d. The tart cherry supplements contained 480 mg of freeze dried Montmorency tart cherry skin powder derived from tart cherry skins obtained after juicing (*CherryPURE™ Freeze Dried Tart Cherry Powder, Shoreline Fruit, LLC, Transverse City, MI, USA*). Prior analytical testing conducted in 2012 by Advanced Laboratories (*Salt Lake City*, *UT*, *USA*) demonstrated that 31 mL (10.5 fl oz) of tart cherry juice provides approximately 600 mg of phenolic compounds and 40 mg of anthocyanins, which is equivalent to consuming 290 mg of *CherryPURE™*. Using the same comparison, the 480 mg *CherryPURE™* supplement provided in the current study would be equivalent to 51.3 mL (17.4 fl oz) of tart cherry juice providing 991 mg of phenolic compounds and 66 mg of anthocyanins. The supplements were prepared for distribution by Shoreline Fruit, LLC and sent to Advanced Laboratories (*Salt Lake City, UT, USA*) to quantify the nutritional contents of the powdered tart cherry supplements. Both supplements were prepared in capsules identical in taste and appearance. The supplements were packaged in generic bottles by Shoreline Fruit, LLC for double blind administration.

### Procedures

#### Dietary inventories

Within the first 7-d of supplementation, subjects were instructed to record all food and fluid intake over a 4-d period (3 weekdays, 1 weekend day). Dietary inventories were then reviewed by a registered dietician and analyzed for average daily energy (total kilocalories), macronutrient (protein, fat, and carbohydrates), and dietary antioxidant (vitamins C and E, and β-carotene) intake using ESHA Food Processor (*Version 8.6*) Nutritional Analysis software (*ESHA Research Inc., Salem, OR, USA*).

#### Anthropometrics and Body composition

At the beginning of every testing session, subjects had their height and body mass measured according to standard procedures using a Healthometer Professional 500KL (*Pelstar LLC, Alsip, IL, USA)* self-calibrating digital scale with an accuracy of ±0.02 kg. Whole body bone density and body composition measures (excluding cranium) were determined with a Hologic Discovery W Dual-Energy X-ray Absorptiometer (DEXA; *Hologic Inc., Waltham, MA, USA*) equipped with APEX Software (*APEX Corporation Software, Pittsburg, PA, USA*) by using procedures previously described [29]. Mean test-retest reliability studies performed on male athletes in our lab with this DEXA machine have revealed mean coefficients of variation for total bone mineral content and total fat free/soft tissue mass of 0.31–0.45 % with a mean intraclass correlation of 0.985 [30]. On the day of each test, the equipment was calibrated following the manufacturer’s guidelines for quality assurance.

#### Muscle soreness perception assessment

Pressure application to the three specified areas of the quadriceps muscle group on each subject’s dominant leg was standardized to 50 N of pressure using a handheld Commander Algometer (*JTECH Medical, Salt Lake City, UT, USA*). The standard amount of pressure was applied to the vastus lateralis at both 25 and 50 % of the distance between the superior border of the patella to the greater trochanter of the femur at the hip and to the vastus medalis at 25 % of the distance between the aforementioned landmarks. These three specific locations were measured and marked with a permanent marker on each subject during the baseline muscle soreness perception measurement before the half-marathon race. The subjects were asked to maintain these three marked locations between testing sessions to avoid error with secondary measurement. The subject was seated in a reclined supine position and given the algometer GPRS sheet to evaluate the perception of muscle soreness at each of the three quadriceps locations. The order of pressure application was standardized across all sessions and subjects: 25 % VM, 25 % VL, and 50 % VL. The 50 N of pressure was applied to a relaxed quadriceps at each of the three locations using the algometer for a period of 3-sec to give the subject enough time to record their soreness evaluation on the GPRS. Perceptions of muscle soreness were recorded by measuring the distance (centimeters) of the participant mark on the GPRS from 0 cm (no pain). Reliability statistical analyses revealed a mean intraclass correlation of 0.909.

#### Blood collection

Subjects donated approximately four teaspoons (20 mL) of venous blood after a 10-h fast from an antecubital vein using standard phlebotomy procedures. Blood samples were collected in two 7.5 mL BD Vacutainer® serum separation tubes (*Becton, Dickinson and Company, Franklin Lakes, NJ, USA)*, left at room temperature for 15-min, and then centrifuged at 3500 rpm for 10-min using a standard, refrigerated (4 °C) bench top Thermo Scientific Heraeus MegaFuge 40R Centrifuge (*Thermo Electron North America LLC, West Palm Beach, FL, USA).* Serum supernatant was removed and stored at −80 °C in polypropylene microcentrifuge tubes for later analysis. The multiple serum microcentrifuge tubes for each subject was allocated for a specific group of assays and thawed only once during analysis. Blood was also collected in a single 3.5 mL BD Vacutainer® containing K_2_ EDTA (*Becton, Dickinson and Company, Franklin Lakes, NJ, USA)*, left at room temperature for 15-min, and refrigerated for approximately 3–4 h before complete blood count analysis.

#### Clinical chemistry analysis

Whole blood samples were analyzed for complete blood count with platelet differentials (hemoglobin, hematocrit, red blood cell counts (RBC), white blood cell counts (WBC), lymphocytes, granulocytes (GRAN), and mid-range absolute count (MID) using a Abbott Cell Dyn 1800 (*Abbott Laboratories, Abbott Park, IL, USA*) automated hematology analyzer. The internal quality control for Abbott Cell Dyn 1800 was performed using three levels of manufacturer control fluids to calibrate acceptable standard deviation (SD) and coefficients of variation (C_V_) values for all aforementioned analytes. Samples were re-run if the observed values were outside control values and/or clinical norms according to standard procedures. Reliability statistical analyses revealed a mean intraclass correlation of 0.729 across all measures. Serum samples were analyzed using a Cobas c111 (*Roche Diagnostics GmbH, Indianapolis, IN, USA*) automated clinical chemistry analyzer that was calibrated according to manufacturer guidelines. This analyzer has been known to be highly valid and reliable in previously published reports [31]. Each serum sample was assayed for a standard partial metabolic panel [(aspartate aminotransferase (AST), alanine aminotransferase (ALT), and total bilirubin)] and clinical markers of protein and fatty acid metabolism [(uric acid, creatinine, blood urea nitrogen (BUN), BUN:creatinine ratio, total protein, and creatine kinase (CK)]. The internal quality control for the Cobas c111 was performed using two levels of manufacturer control fluids to calibrate acceptable SD and C_V_ values for all aforementioned assays. Samples were re-run if the observed values were outside control values and/or clinical norms according to standard procedures. Reliability statistical analyses revealed a mean intraclass correlation of 0.793 across all measures.

#### Markers of anabolic/catabolic hormone status

Serum samples were assayed using standard commercially available enzyme-linked immunosorbent assay kits (ELISAs) for cortisol and testosterone (*ALPCO Diagnostics, Salem, NH, USA*). Serum concentrations were determined calorimetrically using a BioTek ELX-808 Ultramicroplate reader (*BioTek Instruments Inc., Winooski, VT, USA*) at an optical density of 450 nm against a known standard curve using manufacturer recommended procedures. Samples were run in duplicate according to standard procedures. Test to test variability of performing these assays yielded average C_V_ values for the aforementioned markers of: CORT (±6.85 %), and TEST (±4.47 %) with a test retest correlation for the same markers of: CORT (*r =* 0.92), TEST (*r =* 0.98).

#### Markers of oxidative stress

Serum samples were assayed using standard commercially available ELISA kits for Superoxide Dismutase (SOD Activity Assay kit), Total Antioxidant Status (TAS, Antioxidant Assay kit), Thiobarbituric Acid Reactive Substance (TBARS, Malondialdehyde-MDA, TCA method kit) (*Cayman Chemical Company, Ann Arbor, MI, USA*), and Nitrotyrosine (*ALPCO Diagnostics, Salem, NH, USA*). Serum concentrations for SOD and Nitrotyrosine were determined calorimetrically using a BioTek ELX-808 Ultramicroplate reader (*BioTek Instruments Inc., Winooski, VT, USA*) at an optical density of 450 nm against a known standard curve using standard procedures, while TAS serum concentrations were analyzed calorimetrically at 405 nm. Lastly, serum concentrations for TBARS were determined fluorometrically using a SpectraMax Gemini multimode plate reader (*Molecular Devices LLC, Sunnyvale, CA, USA*) at an excitation wavelength of 530 nm and an emission wavelength of 550 nm against a known standard curve using standard procedures. Samples were run in duplicate according to standard procedures. Test to test variability of performing these assays yielded average C_V_ values for the aforementioned markers of: SOD (±8.35 %), TAS (±14.24 %), TBARS (±8.30 %), and NT (±10.03 %) with a test retest correlation for the same markers of: SOD (*r =* 0.83), TAS (*r =* 0.85), TBARS (*r =* 0.94), and NT (*r =* 0.99).

#### Cytokine/Chemokine markers of inflammation

Serum markers of inflammation [(interleukin-1β (IL-1β), IL-2, IL-4, IL-5, IL-6, IL-7, IL-8, IL-10, IL-12p70, IL-13, tumor necrosis factor-α (TNF-α), interferon-γ (IFN-γ), and granulocyte-macrophage colony-stimulating factor (GM-CSF)] were measured by using a commercially available Milliplex MAP 13-Plex Human High Sensitivity T-Cell Magnetic Bead Panel kit (*EMD Millipore Corporation, St. Charles, MO, USA*). A minimum of 100 positive beads for each cytokine/chemokine was acquired with a Luminex MagPix instrument (*Luminex Corporation, Austin, TX, USA*). Samples were run in duplicate according to standard procedures. Test to test variability of performing these assays yielded an average C_V_ value range of ±4.26 to ±6.05 % for the aforementioned markers with an average test retest correlation of *r =* 0.99 for the same markers.

### Statistical analysis

Individual group and time data are presented throughout as means (± SD), while group effects are presented as means (± SEM). All related variables were grouped and analyzed using repeated measures MANOVA in IBM SPSS Statistics Software version 22.0 for Windows (*IBM Corporation, Armonk, NY, USA*). Half-marathon finish time was also used as a covariate in subsequent ANCOVA analyses to determine if previously reported statistical outcomes were attributed to running intensity or to supplementation. Post-hoc LSD pairwise comparisons were used to analyze any significance among groups where needed with Cohen’s d calculations employed to determine effect magnitude. Data were considered statistically significant when the probability of error was less than 0.05 and considered to be trending when the probability of error was between 0.05 and 0.10.

## Results

### Subject characteristics

A total of 27 healthy, endurance trained or triathlete men (*n* = 18) and women (*n* = 9) completed the study protocol. Participant demographic data are presented in Table 1. One-way ANOVA revealed no significant differences (*p* >0.05) in baseline demographic or anthropometric markers.

**Table 1:** Demographics by study group

Variable	Group	Mean	Group (SEM)	*p*-value
N	P	16	n/a	n/a
	TC	11	n/a	
	Total	27	n/a	
Age	P	22.44 ± 4.86	1.214	0.305
	TC	20.82 ± 1.89	0.569	
	Total	21.78 ± 3.95	0.761	
Height (cm)	P	173 ± 11.43	2.851	0.592
	TC	175 ± 8.59	2.589	
	Total	174 ± 10.25	1.972	
Body Mass (kg)	P	65.48 ± 12.07	3.018	0.317
	TC	70.17 ± 11.25	3.392	
	Total	67.39 ± 11.76	2.263	
Baseline HR (bpm)	P	58.50 ± 9.02	2.255	0.703
	TC	59.64 ± 4.46	1.343	
	Total	58.96 ± 58.96	1.426	
BMD (g/cm^2^)	P	1.04 ± 0.11	0.028	0.458
	TC	1.08 ± 0.13	0.038	
	Total	1.06 ± 0.12	0.023	
FFM (kg)	P	48.67 ± 11.32	2.830	0.171
	TC	54.85 ± 11.01	3.319	
	Total	51.19 ± 11.41	2.195	
FM (kg)	P	9.81 ± 3.20	0.801	0.085^§^
	TC	7.76 ± 2.44	0.735	
	Total	2.44 ± 3.04	0.585	
Body Fat (%)	P	16.87 ± 6.40	1.599	0.051^§^
	TC	12.31 ± 4.42	1.333	
	Total	15.01 ± 6.03	1.160	

Mean data expressed as means ± SD. Data represents general study population demographics and anthropometric measures. One-way ANOVA p-levels listed for each variable: § represents *p* <0.10 difference between groups. *HR* heart rate, *BMD* bone mineral density, *LM* lean mass, *FFM* free-fat mass, *FM* fat mass

### Nutritional intake and compliance

Table 2 lists relevant nutrition components analyzed in the 4-d dietary recall. P tended to consume a smaller amount of average daily calories compared to TC (31.0 kcal/kg vs. 37.4 kcal/kg, *p* = 0.094). This differential is likely due dropped subjects (see Fig. 1) causing a greater proportion of females in P (n_f_ = 3/11, 27.3 %) versus TC (n_f_ = 6/16, 37.5 %). When stratifying the statistical dietary analysis by gender within each group, average daily calorie (*p* = 0.44) and dietary carbohydrate (*p* = 0.64) consumption was the same across groups. No other statistically significant interactions were observed across groups with respect to dietary intake.

**Table 2:** Relative dietary analysis by study group

Variable	Group	Mean	Group (SEM)	*p*-value
Average Daily Caloric Consumption (kcal/kg)	P	30.89 ± 8.75	2.19	0.094^§^
	TC	37.71 ± 11.65	3.51	
	Total	33.67 ± 10.40	2.00	
Dietary Protein (g/kg)	P	1.29 ± 0.56	0.14	0.146
	TC	1.62 ± 0.58	0.17	
	Total	1.42 ± 0.58	0.11	
Dietary Carbohydrates (g/kg)	P	3.54 ± 1.50	0.37	0.138
	TC	4.57 ± 2.00	0.60	
	Total	3.96 ± 1.76	0.34	
Dietary Fat (g/kg)	P	1.24 ± 0.49	0.12	0.886
	TC	1.27 ± 0.72	0.22	
	Total	1.25 ± 0.58	0.11	
Dietary Beta-Carotene (mcg/kg)	P	38.01 ± 71.15	17.79	0.611
	TC	54.22 ± 92.29	27.83	
	Total	44.62 ± 79.13	15.23	
Dietary Vitamin C [Ascorbic Acid] (mg/kg)	P	0.92 ± 0.69	0.17	0.277
	TC	1.46 ± 1.78	0.54	
	Total	1.14 ± 1.25	0.24	
Dietary Vitamin E [Alpha-Tocopherol] (mg/kg)	P	0.099 ± 0.095	0.024	0.853
	TC	0.106 ± 0.107	0.032	
	Total	0.102 ± 0.098	0.019	

Mean data expressed as means ± SD. Data represents nutritional analysis from subject 4-d dietary records accounting for subject body mass as a computation of relative dietary components. One-way ANOVA p-levels listed for each variable: § represents *p* <0.10 difference between groups

### Half-marathon performance measures

Table 3 presents half-marathon split and finish times in addition to projected versus actual average race paces. There was no difference in projected race finish times between groups (*p* = 0.304). TC subjects had faster half-marathon split (*p* = 0.002) and race finish times (*p* = 0.001) corresponding to a quicker overall race pace compared to P. The actual race pace was slower compared to the projected race pace in both groups (*p* <0.001), but the difference tended to be smaller (*p* = 0.091) in TC compared to P. Due to the significant difference in race performance, half-marathon finish time was used as a covariate in subsequent ANCOVA analyses to determine if other statistical outcomes were attributed to running intensity or to supplementation.

**Table 3:** Running performance by study group

Variable	Group	Mean	Group (SEM)	*p*-value
½ Marathon Split Time (min)	P	54.30 ± 4.18	1.045	0.002*
	TC	49.03 ± 3.65	1.099	
	Total	52.15 ± 4.71	0.906	
½ Marathon Finish Time (min)	P	118 ± 9.72	2.429	0.001*
	TC	103 ± 9.28	2.798	
	Total	112 ± 11.86	2.283	
½ Marathon Projected Race Pace (min/km)	P	12.00 ± 1.28	0.338	0.304
	TC	11.45 ± 1.45	0.407	
	Total	11.77 ± 1.35	0.264	
½ Marathon Actual Race Pace (min/km)	P	14.48 ± 1.19	0.293	0.002*
	TC	12.70 ± 1.14	0.354	
	Total	13.76 ± 1.45	0.230	

Mean data expressed as means ± SD. Data represents the half-marathon performance measures. Half-marathon projected race pace figures were calculated based upon subjects’ self-reported previous endurance running race performances. The overall MANOVA analysis revealed overall Wilks’ Lambda time (*p* <0.001) and group x time (*p* = 0.091). Univariate ANOVA p-levels from the MANOVA analysis are presented for both pacing variables. One-way ANOVA p-levels listed for each timing variable: * represents *p* <0.05 difference between groups

### Markers of mechanical damage and physiological stress

**Fig. 3:**
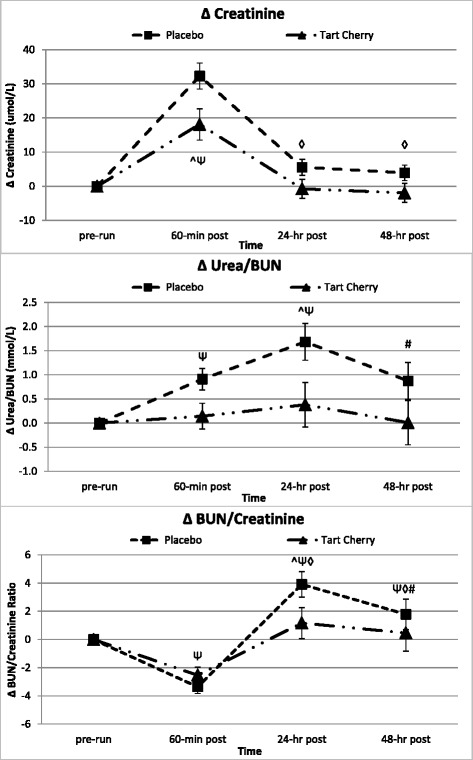
Secondary indices of muscle damage and protein catabolism. Data expressed as means ± SE and significance indicated by the following super/subscripts: * indicates *p* <0.05 p-level significance, § indicates *p* <0.10 p-level significance. LSD post hoc analysis is indicated by the following superscripts: ^ represents *p* <0.05 difference between groups, Ψ represents *p* <0.05 difference from pre-run, ◊ represents *p* <0.05 difference from 60-min post, # represents *p* <0.05 difference from 24-hr post

**Fig. 4:**
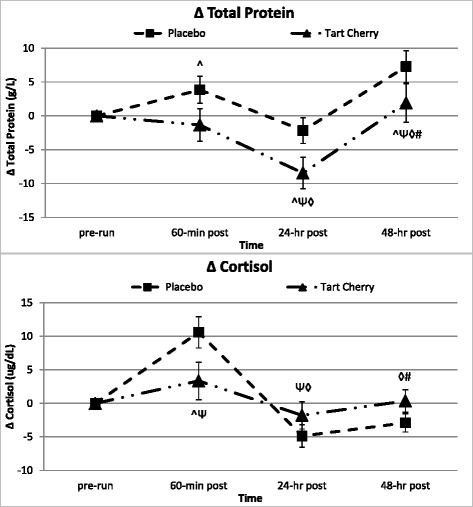
Markers of protein catabolism and physiological stress. Data expressed as means ± SE and significance indicated by the following super/subscripts: * indicates *p* <0.05 p-level significance, § indicates *p* <0.10 p-level significance. LSD post hoc analysis is indicated by the following superscripts: ^ represents *p* <0.05 difference between groups, Ψ represents *p* <0.05 difference from pre-run, ◊ represents *p* <0.05 difference from 60-min post, # represents *p* <0.05 difference from 24-hr post

Table 4 presents the serum mechanical damage and physiological stress marker data. Serum creatinine and urea/BUN makers increased on average 19 and 21 %, respectively, over pre-run values during the recovery in P, but only 6 and 3 % in TC. Serum total protein content increased on average 4 % over pre-run values during the recovery in P, but decreased 3 % below pre-run in TC. Significant (or trends approaching significance) changes across groups and group differences over time for creatinine (*p* = 0.047, group *p* = 0.007), urea/BUN (*p* = 0.048, group *p* = 0.004), and total protein (*p* = 0.081, group *p* = 0.060) were further supported by ANCOVA analyses accounting for running intensity. Subsequent post-hoc analysis indicated a significantly attenuated serum creatinine level 60-min post-run and a mitigated urea/BUN response in TC compared to P 24-h post-run (see Fig. 3). The total protein response never increased above pre-run levels over the 48-h recovery in TC compared to significant elevations 60-min and 48-h post-run in P (see Fig. 4).

**Table 4:** Markers of muscle catabolism, Secondary muscle damage, and Physiological stress

Variable	Group	Baseline	Pre-Run	60-min Post	24-hr Post	48-hr Post	Group Mean	*p*-value (GG)	*p*-value (WSC)	RFT Covariate *p*-value (WSC)
AST (U/L)	P	32.69 ± 33.67	26.62 ± 14.97	37.20 ± 17.36	50.52 ± 22.96	43.33 ± 16.68	38.07 ± 3.98	*G =* 0.911		*G =* 0.402
	TC	29.46 ± 10.54	26.30 ± 7.48	36.96 ± 9.68	49.97 ± 31.12	44.14 ± 24.60	37.37 ± 4.80	*T =* 0.002*	*T*_*L*_ *=* 0.005*	*T*_*q*_ *=* 0.593
	Time Mean	31.08 ± 5.27	26.46 ± 2.45	37.08 ± 2.89^†Ψ^	50.25 ± 5.20^†Ψ◊^	43.74 ± 3.96^†Ψ#^		*G X T =* 0.859	*G X T*_*q*_ *=* 0.740	*G X T*_*L*_ *=* 0.707
ALT (U/L)	P	22.09 ± 13.55	20.32 ± 7.15	23.65 ± 8.04	26.42 ± 7.36	28.53 ± 8.02	24.20 ± 2.15	*G =* 0.576		*G =* 0.057^§^
	TC	23.06 ± 8.22	23.13 ± 10.39	AM ± 10.22	27.94 ± 14.22	30.07 ± 14.80	26.11 ± 2.59	*T =* 0.008*	*T*_*L*_ *=* 0.006*	*T*_*q*_ *=* 0.677
	Time Mean	22.57 ± 2.29	21.73 ± 1.68	25.01 ± 1.76^Ψ^	27.18 ± 2.09^Ψ◊^	29.30 ± 2.20^†Ψ◊#^		*G X T =* 0.842	*G X T*_*q*_ *=* 0.603	*G X T*_*L*_ *=* 0.869
Total Billirubin (umol/L)	P	9.01 ± 2.87	8.15 ± 3.03	13.03 ± 4.68	8.44 ± 4.06	7.65 ± 3.45	9.25 ± 0.76	*G =* 0.756		*G =* 0.614
	TC	8.71 ± 4.64	7.60 ± 3.75	11.65 ± 4.50	8.99 ± 4.22	7.46 ± 2.75	8.88 ± 0.91	*T <*0.001*	*T*_*q*_ *=* 0.001*	*T*_*q*_ *=* 0.484
	Time Mean	8.86 ± 0.72	7.88 ± 0.65	12.34 ± 0.90^†Ψ^	8.71 ± 0.81^◊^	7.55 ± 0.62^◊^		*G X T =* 0.699	*G X T*_*L*_ *=* 0.694	*G X T*_*q*_ *=* 0.591
Urea/BUN (mmol/L)	P	4.75 ± 1.08	5.45 ± 1.36	6.36 ± 1.14	7.13 ± 1.15	6.33 ± 1.44	6.00 ± 0.21	*G =* 0.857		*G =* 0.426
	TC	5.11 ± 0.69	6.02 ± 1.17	6.16 ± 1.01	6.40 ± 1.44	6.03 ± 1.58	5.94 ± 0.26	*T <*0.001*	*T*_*L*_ *<*0.001*	*T*_*L*_ *=* 0.026*
	Time Mean	4.93 ± 0.19	5.74 ± 0.25^†^	6.26 ± 0.21^†Ψ^	6.77 ± 0.25^†Ψ^	6.18 ± 0.29^†#^		*G X T =* 0.144	*G X T*_*L*_ *=* 0.095^§^	*G X T*_*L*_ *=* 0.014*
Creatinine (umol/L)	P	71.71 ± 15.07	74.29 ± 11.54	106.56 ± 15.45^†Ψ^	79.85 ± 10.88^†◊^	78.20 ± 11.17^†^	82.12 ± 3.12	*G =* 0.651		*G =* 0.522
	TC	77.20 ± 15.93	82.29 ± 13.96	100.44 ± 25.52^†Ψ^	81.54 ± 15.08^◊^	80.34 ± 12.72	84.36 ± 3.77	*T <*0.001*	*T*_*q*_ *<*0.001*	*T*_*L*_ *=* 0.007*
	Time Mean	74.45 ± 3.02	78.29 ± 2.46	103.50 ± 3.94^†Ψ^	80.70 ± 2.49^†◊^	79.27 ± 2.31^◊^		*G X T =* 0.087^§^	*G X T*_*L*_ *=* 0.246	*G X T*_*L*_ *=* 0.010*
BUN/Creatinine Ratio	P	16.60 ± 3.05	18.58 ± 5.58	15.22 ± 4.38	22.49 ± 4.61	20.36 ± 5.29	18.65 ± 0.90	*G =* 0.589		*G =* 0.329
	TC	16.97 ± 3.87	18.33 ± 3.50	15.80 ± 3.53	19.50 ± 3.61	18.79 ± 5.26	17.88 ± 1.09	*T <*0.001*	*T*_*L*_ *=* 0.001*	*T*_*q*_ *=* 0.240
	Time Mean	16.75 ± 3.34	18.48 ± 4.77	15.46 ± 3.99^Ψ^	21.27 ± 4.41^†Ψ◊^	19.72 ± 5.23^†◊#^		*G X T =* 0.158	*G X T*_*L*_ *=* 0.140	*G X T*_*L*_ *=* 0.103^§^
Uric Acid (umol/L)	P	271 ± 46	292 ± 44	373 ± 48	321 ± 48	308 ± 47	313 ± 11	*G =* 0.789		*G =* 0.724
	TC	290 ± 50	308 ± 52	364 ± 64	326 ± 82	302 ± 65	318 ± 14	*T <*0.001*	*T*_*q*_ *<*0.001*	*T*_*L*_ *=* 0.014*
	Time Mean	280 ± 9	300 ± 9^†^	369 ± 11^†Ψ^	324 ± 13^†Ψ◊^	305 ± 11^†◊#^		*G X T =* 0.444	*G X T*_*L*_ *=* 0.188	*G X T*_*L*_ *=* 0.015*
CK (U/L)	P	606 ± 1696	276 ± 510	532 ± 627	907 ± 683	593 ± 525	583 ± 144	*G =* 0.626		*G =* 0.806
	TC	298 ± 317	228 ± 191	474 ± 253	870 ± 771	490 ± 395	472 ± 173	*T =* 0.036*	*T*_*L*_ *=* 0.139	*T*_*q*_ *=* 0.836
	Time Mean	452 ± 260	252 ± 81	503 ± 100^Ψ^	889 ± 141^Ψ◊^	541 ± 93^Ψ#^		*G X T =* 0.680	*G X T*_*q*_ *=* 0.416	*G X T*_*q*_ *=* 0.599
Total Protein (mmol/L)	P	67.51 ± 8.56	72.93 ± 5.41^†^	76.78 ± 4.95^†^	70.77 ± 5.35^◊^	80.21 ± 6.48^†Ψ#^	73.64 ± 0.87	*G =* 0.746		*G =* 0.846
	TC	68.70 ± 8.06	76.28 ± 4.78^†^	74.94 ± 8.85^†^	67.83 ± 4.52^◊^	78.20 ± 6.84^†#^	73.19 ± 1.05	*T <* 0.001*	*T*_*L*_ *<*0.001*	*T*_*L*_ *=* 0.008*
	Time Mean	68.11 ± 1.64	74.61 ± 1.01^†^	75.86 ± 1.33^†^	69.30 ± 0.99^Ψ◊^	79.20 ± 1.30^†Ψ◊#^		*G X T =* 0.316	*G X T*_*L*_ *=* 0.066^§^	*G X T*_*L*_ *=* 0.004*

Individual group and time data expressed as means ± SD, while group effects are presented as means ± SEM. Data represents the response to muscle catabolism, mechanical damage, and physiological stress at each testing session during the 10 day intervention. MANOVA analysis revealed overall Wilks’ Lambda time (*p* <0.001) and group x time (*p* = 0.504). Univariate ANOVA p-levels from MANOVA analysis are presented for each variable. Univariate ANOVA p-levels are listed first by the Greenhouse-Geisser (GG) analysis and then by the within-subjects contrasts (WSC) to demonstrate the potential shape of the time or group x time interaction with significance indicated by the following super/subscripts: *indicates *p* <0.05 p-level significance, §indicates *p* <0.10 p-level significance. LSD post hoc analysis is indicated by the following superscripts: † represents *p* <0.05 difference from baseline value, Ψ represents *p* <0.05 difference from pre-run, ◊ represents *p* <0.05 difference from 60-min post, # represents *p* <0.05 difference from 24-hr post. *AST* aspartate aminotransferase, *ALT* alanine aminotransferase, *BUN* blood urea nitrogen, *CK* creatine kinase, *RFT* race finish time, *G* group p-level, *T* time p-level, *G x T* interaction, *q* quadratic p-level, *L* linear p-level

### Anabolic/catabolic hormone response markers

Table 5 demonstrates the serum testosterone and cortisol hormone marker response. Testosterone and cortisol demonstrated significant changes over time and from baseline, peaking (elevated or depressed) 60-min post-run. Significant group differences over time and from pre-run levels were reported for serum cortisol (*p* = 0.012, delta *p* = 0.016). Serum cortisol levels 60-min post-run increased 44 % over pre-run values in P, but only 15 % in TC. Subsequent post-hoc analysis indicated significantly attenuated serum cortisol levels in TC compared to P 60-min and 24-h post-run (see Fig. 4). These results were supported when accounting for differences in running intensity.

**Table 5:** Anabolic/Catabolic hormone response

Variable	Group	Baseline	Pre-Run	60-min Post	24-hr Post	48-hr Post	Group Mean	*p*-value (GG)	*p*-value (WSC)	RFT Covariate *p*-value (WSC)
Cortisol (ug/dL)	P	20.92 ± 7.21	24.04 ± 5.16	34.63 ± 10.14^^†Ψ^	19.17 ± 4.87^Ψ◊^	21.14 ± 5.83^◊^	23.98 ± 1.23	*G =* 0.408		*G =* 0.868
	TC	21.34 ± 6.68	22.14 ± 7.35	25.48 ± 7.45	20.36 ± 5.93^◊^	22.50 ± 4.77	22.36 ± 1.48	*T <*0.001*	*T*_*q*_ *<*0.001*	*T*_*L*_ *=* 0.259
	Time Mean	21.13 ± 1.37	23.09 ± 1.20	30.06 ± 1.80^†Ψ^	19.76 ± 1.04^Ψ◊^	21.82 ± 1.06^◊#^		*G X T =* 0.005*	*G X T*_*q*_ *=* 0.012*	*G X T*_*q*_ *=* 0.030*
Testosterone (ng/mL)	P	7.22 ± 3.90	6.77 ± 3.55	6.34 ± 3.55	6.48 ± 3.32	6.74 ± 3.41	6.71 ± 0.88	*G =* 0.745		*G =* 0.497
	TC	6.65 ± 4.24	6.52 ± 3.89	5.59 ± 3.48	6.21 ± 3.75	6.31 ± 3.69	6.26 ± 1.06	*T =* 0.058^§^	*T*_*q*_ *=* 0.042*	*T*_*q*_ *=* 0.026*
	Time Mean	6.94 ± 0.79	6.64 ± 0.72	5.96 ± 0.69^†Ψ^	6.34 ± 0.69	6.53 ± 0.69		*G X T =* 0.848	*G X T*_*L*_ *=* 0.881	*G X T*_*L*_ *=* 0.171
Test/Cort Ratio	P	0.037 ± 0.026	0.030 ± 0.018	0.019 ± 0.010	0.035 ± 0.024	0.034 ± 0.020	0.031 ± 0.005	*G =* 0.874		*G =* 0.546
	TC	0.035 ± 0.023	0.035 ± 0.023	0.026 ± 0.023	0.034 ± 0.024	0.030 ± 0.021	0.032 ± 0.006	*T =* 0.001*	*T*_*q*_ *=* 0.009*	*T*_*q*_ *=* 0.588
	Time Mean	0.036 ± 0.024	0.032 ± 0.020	0.022 ± 0.016^†Ψ^	0.035 ± 0.023^◊^	0.032 ± 0.020^◊^		*G X T =* 0.327	*G X T*_*q*_ *=* 0.101^§^	*G X T*_*q*_ *=* 0.290

Individual group and time data expressed as means ± SD, while group effects are presented as means ± SEM. Data represents the stress and sex hormone response at each testing session during the 10 day intervention. MANOVA analysis revealed overall Wilks’ Lambda time (*p* <0.001) and group x time (*p* = 0.102). Univariate ANOVA p-levels from MANOVA analysis are presented for each variable. Univariate ANOVA p-levels are listed first by the Greenhouse-Geisser (GG) analysis and then by the within-subjects contrasts (WSC) to demonstrate the potential shape of the time or group x time interaction with significance indicated by the following super/subscripts: * indicates *p* <0.05 p-level significance, § indicates *p* <0.10 p-level significance. LSD post hoc analysis is indicated by the following superscripts: ^ represents *p* <0.05 difference between groups, † represents *p* <0.05 difference from baseline value, Ψ represents *p* <0.05 difference from pre-run, ◊ represents *p* <0.05 difference from 60-min post, # represents *p* <0.05 difference from 24-hr post. *Cort/Test* Cortisol/Testosterone ratio, *RFT* race finish time, *G* group p-level, *T* time p-level, *G x T* interaction, *q* quadratic p-level, *L* linear p-level

### Markers of free radical production and oxidative stress

**Fig. 5:**
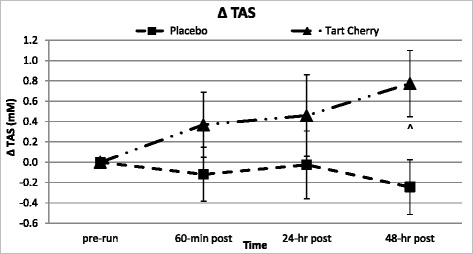
Changes in antioxidant activity with supplementation and endurance exercise. Data expressed as means ± SE and significance indicated by the following super/subscripts: * indicates *p* <0.05 p-level significance, § indicates *p* <0.10 p-level significance. LSD post hoc analysis is indicated by the following superscripts: ^ represents *p* <0.05 difference between groups, Ψ represents *p* <0.05 difference from pre-run, ◊ represents *p* <0.05 difference from 60-min post, # represents *p* <0.05 difference from 24-hr post

Table 6 shows the response of free radical production and oxidative stress markers. None of the measures for free radical production or oxidative stress demonstrated significant changes over time. Serum TAS levels tended to be different between groups over time (*p* = 0.089), which was supported when accounting for running intensity differences. Serum TAS levels decreased 1–8 % from pre-run levels in P over the 48-h recovery, but increased 15–31 % in TC (*p* = 0.046). Post-hoc analysis revealed a linear increase in TC serum TAS activity from pre-run levels that was statistically different from P and pre-run values at 48-h of recovery (see Fig. 5).

**Table 6:** Markers of free radical production and oxidative stress

Variable	Group	Baseline	Pre-Run	60-min Post	24-hr Post	48-hr Post	Group Mean	p-value (GG)	*p*-value (WSC)	RFT Covariate *p*-value (WSC)
Nitrotyrosine (nM)	P	279 ± 305	214 ± 164	231 ± 180	193 ± 139	198 ± 156	223 ± 71	*G =* 0.493		*G =* 0.852
	TC	305 ± 425	284 ± 382	319 ± 436	260 ± 352	302 ± 435	294 ± 85	*T =* 0.177	*T*_*L*_ *=* 0.183	*T*_*q*_ *=* 0.667
	Time Mean	292 ± 70	249 ± 53	275 ± 60^Ψ^	227 ± 48^Ψ◊^	250 ± 58^◊^		*G X T =* 0.699	*G X T*_*L*_ *=* 0.619	*G X T*_*L*_ *=* 0.597
TBARS (uM)	P	8.24 ± 4.62	7.59 ± 2.54	7.01 ± 4.24	7.35 ± 3.52	7.73 ± 3.39	7.59 ± 0.71	*G =* 0.484		*G =* 0.840
	TC	8.34 ± 4.06	7.94 ± 4.08	7.91 ± 3.46	8.93 ± 4.52	8.79 ± 4.09	8.38 ± 0.86	*T =* 0.690	*T*_*q*_ *=* 0.332	*T*_*q*_ *=* 0.462
	Time Mean	8.29 ± 0.86	7.77 ± 0.64	7.46 ± 0.77	8.14 ± 0.77	8.26 ± 0.72		*G X T =* 0.783	*G X T*_*L*_ *=* 0.547	*G X T*_*L*_ *=* 0.520
TAS (mM)	P	3.13 ± 0.85	3.20 ± 0.88^	3.08 ± 0.89	3.18 ± 1.08	2.96 ± 0.95	3.11 ± 0.17	*G =* 0.476		*G =* 0.381
	TC	2.99 ± 0.84	2.50 ± 0.97	2.87 ± 1.07	2.96 ± 1.26	3.27 ± 0.87^Ψ^	2.92 ± 0.20	*T =* 0.713	*T*_*q*_ *=* 0.321	*T*_*L*_ *=* 0.436
	Time Mean	3.06 ± 0.17	2.85 ± 0.18	2.98 ± 0.19	3.07 ± 0.23	3.12 ± 0.18		*G X T =* 0.239	*G X T*_*q*_ *=* 0.089^§^	*G X T*_*q*_ *=* 0.089^§^
SOD (U/mL)	P	0.49 ± 0.09	0.52 ± 0.11	0.50 ± 0.12	0.49 ± 0.08	0.49 ± 0.14	0.50 ± 0.02	*G =* 0.198		*G =* 0.285
	TC	0.45 ± 0.10	0.46 ± 0.16	0.47 ± 0.15	0.42 ± 0.15	0.48 ± 0.10	0.46 ± 0.02	*T =* 0.687	*T*_*L*_ *=* 0.756	*T*_*L*_ *=* 0.155
	Time Mean	0.47 ± 0.02	0.49 ± 0.03	0.49 ± 0.03	0.46 ± 0.02	0.48 ± 0.03		*G X T =* 0.808	*G X T*_*L*_ *=* 0.564	*G X T*_*L*_ *=* 0.656

Individual group and time data expressed as means ± SD, while group effects are presented as means ± SEM. Data represents the response to reactive oxygen and nitrogen species production in addition to antioxidant activity at each testing session during the 10 day intervention. MANOVA analysis revealed overall Wilks’ Lambda time (*p* <0.001) and group x time (*p* = 0.684). Univariate ANOVA p-levels from MANOVA analysis are presented for each variable. Univariate ANOVA p-levels are listed first by the Greenhouse-Geisser (GG) analysis and then by the within-subjects contrasts (WSC) to demonstrate the potential shape of the time or group x time interaction with significance indicated by the following super/subscripts: § indicates *p* <0.10 p-level significance. LSD post hoc analysis is indicated by the following superscripts: ^ represents *p* <0.05 difference between groups, Ψ represents *p* <0.05 difference from pre-run, ◊ represents *p* <0.05 difference from 60-min post. *TBARS* thiobarbituric acid reactive substances, *TAS* total antioxidant status, *SOD* superoxide dismutase, *RFT* race finish time, *G* group p-level, *T* time p-level, *G x T* interaction, *q* quadratic p-level, *L* linear p-level

### Inflammatory response markers

**Fig. 6:**
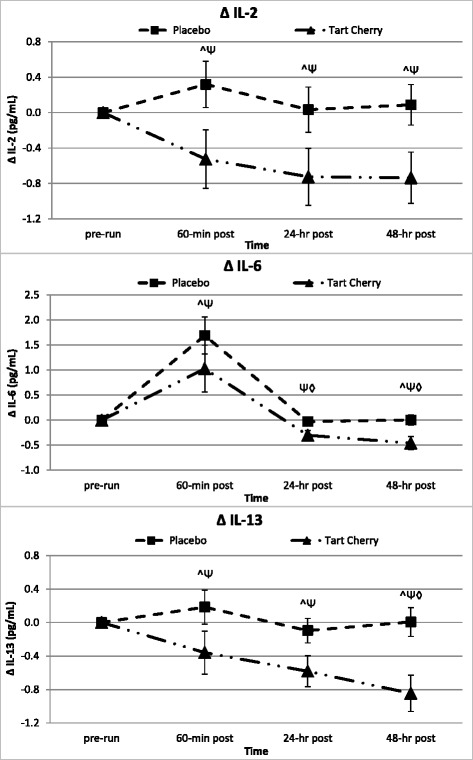
Influence of supplementation and endurance exercise on markers of the inflammatory and anti-inflammatory response. Data expressed as means ± SE and significance indicated by the following super/subscripts: * indicates *p* <0.05 p-level significance, § indicates *p* <0.10 p-level significance. LSD post hoc analysis is indicated by the following superscripts: ^ represents *p* <0.05 difference between groups, Ψ represents *p* <0.05 difference from pre-run, ◊ represents *p* <0.05 difference from 60-min post, # represents *p* <0.05 difference from 24-hr post

Table 7 shows the serum inflammatory cytokine and chemokine marker response. Accounting for running intensity differences, both IL-2 (*p* = 0.089) and IL-6 (*p* = 0.064) measures tended to be different between groups over time. Serum IL-2 levels increased 0.2 % from pre-run levels throughout recovery in P, but decreased 28 % in TC. Further, serum IL-6 levels increased 64 % from pre-run in P, but only 17 % in TC. Delta post-hoc analyses demonstrated significant attenuation of serum IL-6 measures 60-min post-run in TC compared to P (104 % TC increase vs. 210 % P increase). Serum IL-2 significantly decreased in TC compared to P and pre-run measures over the 48-h recovery (see Fig. 6).

**Table 7:** Pro-inflammatory cytokines and chemokines

Variable	Group	Baseline	Pre-Run	60-min Post	24-hr Post	48-hr Post	Group Mean	*p*-value (GG)	*p*-value (WSC)	RFT Covariate *p*-value (WSC)
TNF-α (pg/mL)	P	2.34 ± 1.23	2.74 ± 1.22	3.02 ± 1.55	2.48 ± 1.04	2.49 ± 0.90	2.61 ± 0.26	*G =* 0.922		*G =* 0.654
	TC	2.49 ± 0.98	2.82 ± 0.90	2.93 ± 1.36	2.60 ± 0.91	2.42 ± 0.82	2.65 ± 0.31	*T =* 0.003*	*T*_*q*_ *=* 0.003*	*T*_*L*_ *=* 0.156
	Time Mean	2.40 ± 1.12	2.77 ± 1.08^†^	2.98 ± 1.45^†^	2.53 ± 0.97^Ψ◊^	2.46 ± 0.85^Ψ◊^		*G X T =* 0.779	*G X T*_*L*_ *=* 0.504	*G X T*_*L*_ *=* 0.151
IFN-γ (pg/mL)	P	7.67 ± 6.52	8.02 ± 7.77	8.73 ± 9.49	7.36 ± 7.15	7.75 ± 7.78	7.91 ± 2.55	*G =* 0.382		*G =* 0.570
	TC	10.96 ± 11.44	10.71 ± 9.14	11.51 ± 12.50	12.47 ± 17.61	11.68 ± 15.42	11.47 ± 3.08	*T =* 0.604	*T*_*q*_ *=* 0.184	*T*_*L*_ *=* 0.424
	Time Mean	9.01 ± 8.81	9.12 ± 8.29	9.86 ± 10.67	9.44 ± 12.46	9.35 ± 11.41		*G X T =* 0.388	*G X T*_*L*_ *=* 0.456	*G X T*_*q*_ *=* 0.461
IL-1β (pg/mL)	P	0.67 ± 0.15	0.85 ± 0.21	0.84 ± 0.19	0.75 ± 0.20	0.69 ± 0.16	0.76 ± 0.08	*G =* 0.059^§^		*G =* 0.058^§^
	TC	0.93 ± 0.51	1.12 ± 0.58	1.05 ± 0.39	0.98 ± 0.44	0.93 ± 0.40	1.00 ± 0.09	*T <*0.001*	*T*_*q*_ *<*0.001*	*T*_*L*_ *=* 0.559
	Time Mean	0.78 ± 0.36	0.96 ± 0.42^†^	0.92 ± 0.30^†^	0.85 ± 0.33^Ψ◊^	0.79 ± 0.30^Ψ◊^		*G X T =* 0.860	*G X T*_*q*_ *=* 0.662	*G X T*_*L*_ *=* 0.465
IL-2 (pg/mL)	P	1.18 ± 0.96	1.33 ± 1.10	1.51 ± 1.38	1.23 ± 0.85	1.27 ± 0.94	1.30 ± 0.28	*G =* 0.939		*G =* 0.786
	TC	1.17 ± 1.20	1.64 ± 2.18	1.32 ± 0.99	1.12 ± 1.01	1.11 ± 1.06	1.27 ± 0.34	*T =* 0.070^§^	*T*_*q*_ *=* 0.001*	*T*_*L*_ *=* 0.293
	Time Mean	1.17 ± 1.04	1.46 ± 1.60^†^	1.43 ± 1.22^†^	1.18 ± 0.90^◊^	1.21 ± 0.97^◊^		*G X T =* 0.290	*G X T*_*L*_ *=* 0.195	*G X T*_*L*_ *=* 0.089^§^
IL-6 (pg/mL)	P	0.63 ± 0.54^	0.75 ± 0.53^	2.33 ± 1.38^†Ψ^	0.69 ± 0.44^^◊^	0.68 ± 0.43	1.02 ± 0.19	*G =* 0.017*		*G =* 0.509
	TC	0.94 ± 1.00	1.14 ± 1.27^†^	2.32 ± 1.69^†Ψ^	0.89 ± 0.97^Ψ◊^	0.79 ± 0.76^Ψ^	1.21 ± 0.23	*T <*0.001*	*T*_*q*_ *<*0.001*	*T*_*q*_ *=* 0.648
	Time Mean	0.76 ± 0.76	0.91 ± 0.90^†^	2.33 ± 1.48^†Ψ^	0.77 ± 0.69^Ψ◊^	0.72 ± 0.58^Ψ◊^		*G X T =* 0.550	*G X T*_*L*_ *=* 0.053^§^	*G X T*_*L*_ *=* 0.064^§^
IL-8 (pg/mL)	P	2.74 ± 1.58	2.84 ± 1.29	6.21 ± 3.51	2.94 ± 1.24	2.52 ± 1.01	3.45 ± 0.4	*G =* 0.002*		*G =* 0.637
	TC	3.31 ± 1.87	3.24 ± 1.71	5.39 ± 2.78	3.26 ± 2.08	2.74 ± 1.47	3.59 ± 0.49	*T <*0.001*	*T*_*q*_ *<*0.001*	*T*_*q*_ *=* 0.185
	Time Mean	2.98 ± 1.69	3.00 ± 1.46	5.88 ± 3.20^†^	3.07 ± 1.61^◊^	2.61 ± 1.20^Ψ◊#^		*G X T =* 0.287	*G X T*_*q*_ *=* 0.269	*G X T*_*q*_ *=* 0.166
IL-12p70 (pg/mL)	P	1.79 ± 1.83	1.84 ± 1.75	1.95 ± 1.93	1.70 ± 1.56	1.81 ± 1.93	1.82 ± 0.42	*G =* 0.009*		*G =* 0.706
	TC	1.54 ± 1.78	1.59 ± 1.52	1.75 ± 1.93	1.34 ± 1.29	1.27 ± 1.12	1.50 ± 0.51	*T =* 0.012*	*T*_*q*_ *=* 0.008*	*T*_*q*_ *=* 0.893
	Time Mean	1.69 ± 1.78	1.74 ± 1.63	1.87 ± 1.89^†^	1.55 ± 1.44^Ψ◊^	1.59 ± 1.65^Ψ◊^		*G X T =* 0.310	*G X T*_*q*_ *=* 0.124	*G X T*_*q*_ *=* 0.167

Individual group and time data expressed as means ± SD, while group effects are presented as means ± SEM. Data represents the pro-inflammatory response at each testing session during the 10 day intervention. MANOVA analysis revealed overall Wilks’ Lambda time (*p* <0.001) and group x time (*p* = 0.302). Univariate ANOVA p-levels from MANOVA analysis are presented for each variable. Univariate ANOVA p-levels are listed first by the Greenhouse-Geisser (GG) analysis and then by the within-subjects contrasts (WSC) to demonstrate the potential shape of the time or group x time interaction with significance indicated by the following super/subscripts: * indicates *p* <0.05 p-level significance, § indicates *p* <0.10 p-level significance. LSD post hoc analysis is indicated by the following superscripts: ^ represents *p* <0.05 difference between groups, † represents *p* <0.05 difference from baseline value, Ψ represents *p* <0.05 difference from pre-run, ◊ represents *p* <0.05 difference from 60-min post, # represents *p* <0.05 difference from 24-hr post. *TNF-α* tumor necrosis factor alpha, *IFN-γ* interferon gamma, *IL* interleukin, *RFT* race finish time, *G* group p-level, *T* time p-level, *G x T* interaction, *q* quadratic p-level, *L* linear p-level

### Anti-inflammatory response markers

Table 8 presents the serum anti-inflammatory cytokine marker results. Accounting for running intensity, serum IL-13 levels were significantly different across groups (*p* = 0.031) and tended to be different between groups over time (*p* = 0.053). Serum IL-13 markers decreased 6 % on average from pre-run over the 48-h recovery in P, compared to a 13 % decrease in TC. Specifically, at 60-min post-run, serum IL-13 markers increased 5 % over pre-run values in P, but actually decreased 7 % below pre-run in TC. The serum IL-13 group (*p* = 0.029) and group by time (*p* = 0.014) differences from pre-run levels over the recovery period were also significant (see Fig. 6).

**Table 8:** Anti-inflammatory cytokines

Variable	Group	Baseline	Pre-Run	60-min Post	24-hr Post	48-hr Post	Group Mean	p-value (GG)	p-value (WSC)	RFT Covariate *p*-value (WSC)
IL-4 (pg/mL)	P	4.57 ± 2.02	5.51 ± 2.31	5.20 ± 2.15	4.02 ± 1.49	4.21 ± 1.63	4.70 ± 1.28	*G =* 0.304		*G =* 0.165
	TC	6.93 ± 8.98	8.71 ± 11.72	6.65 ± 6.37	5.41 ± 5.13	6.38 ± 7.03	6.82 ± 1.55	*T =* 0.012*	*T*_*q*_ *=* 0.012*	*T*_*L*_ *=* 0.309
	Time Mean	5.53 ± 5.89	6.82 ± 7.64	5.79 ± 4.34	4.59 ± 3.45^Ψ◊^	5.09 ± 4.66^Ψ◊#^		*G X T =* 0.320	*G X T*_*q*_ *=* 0.189	*G X T*_*L*_ *=* 0.189
IL-5 (pg/mL)	P	0.57 ± 0.37	0.63 ± 0.42	0.66 ± 0.47	0.66 ± 0.46	0.61 ± 0.40	0.62 ± 0.14	*G =* 0.102^§^		*G =* 0.013*
	TC	0.96 ± 0.75	1.01 ± 0.73	1.07 ± 0.76	1.01 ± 0.80	0.87 ± 0.59	0.98 ± 0.16	*T =* 0.093^§^	*T*_*q*_ *=* 0.013*	*T*_*q*_ *=* 0.452
	Time Mean	0.73 ± 0.58	0.78 ± 0.59	0.82 ± 0.62	0.80 ± 0.63	0.71 ± 0.49^Ψ◊#^		*G X T =* 0.542	*G X T*_*L*_ *=* 0.185	*G X T*_*L*_ *=* 0.205
IL-7 (pg/mL)	P	3.75 ± 1.63	3.37 ± 1.57	4.66 ± 2.61	3.72 ± 1.81	3.47 ± 1.15	3.80 ± 0.43	*G =* 0.295		*G =* 0.427
	TC	4.66 ± 2.54	4.27 ± 1.84	5.12 ± 2.22	4.55 ± 1.69	4.01 ± 1.42	4.52 ± 0.52	*T <*0.001*	*T*_*q*_ *=* 0.010*	*T*_*L*_ *=* 0.845
	Time Mean	4.12 ± 2.06	3.74 ± 1.71^†^	4.85 ± 2.42^†Ψ^	4.06 ± 1.78^◊^	3.69 ± 1.27^◊#^		*G X T =* 0.708	*G X T*_*L*_ *=* 0.492	*G X T*_*L*_ *=* 0.485
IL-10 (pg/mL)	P	2.91 ± 3.13	2.77 ± 1.89	24.17 ± 26.99	2.77 ± 1.78	2.68 ± 1.68	7.06 ± 1.36	*G =* 0.683		*G =* 0.948
	TC	3.31 ± 2.74	3.99 ± 5.07	17.16 ± 18.60	3.66 ± 3.69	2.79 ± 1.98	6.18 ± 1.64	*T =* 0.001*	*T*_*q*_ *=* 0.001*	*T*_*q*_ *=* 0.691
	Time Mean	3.07 ± 2.93	3.27 ± 3.51	21.31 ± 23.79^†Ψ^	3.13 ± 2.70^◊^	2.72 ± 1.77^◊^		*G X T =* 0.421	*G X T*_*q*_ *=* 0.495	*G X T*_*L*_ *=* 0.509
IL-13 (pg/mL)	P	1.52 ± 0.95	1.77 ± 1.14	1.86 ± 1.27	1.55 ± 1.00	1.58 ± 0.86	1.65 ± 0.36	*G =* 0.091^§^		*G =* 0.031*
	TC	2.56 ± 2.01	2.96 ± 2.37	2.74 ± 1.75	2.56 ± 2.00	2.40 ± 1.66	2.65 ± 0.43	*T =* 0.006*	*T*_*q*_ *=* 0.003*	*T*_*q*_ *=* 0.027*
	Time Mean	1.94 ± 1.53	2.25 ± 1.80^†^	2.22 ± 1.52^†^	1.96 ± 1.54^Ψ◊^	1.91 ± 1.29^Ψ◊^		*G X T =* 0.538	*G X T*_*L*_ *=* 0.236	*G X T*_*q*_ *=* 0.053^§^

Individual group and time data expressed as means ± SD, while group effects are presented as means ± SEM. Data represents the anti-inflammatory response at each testing session during the 10 day intervention. MANOVA analysis revealed overall Wilks’ Lambda time (*p* <0.001) and group x time (*p* = 0.447). Univariate ANOVA p-levels from MANOVA analysis are presented for each variable. Univariate ANOVA p-levels are listed first by the Greenhouse-Geisser (GG) analysis and then by the within-subjects contrasts (WSC) to demonstrate the potential shape of the time or group x time interaction with significance indicated by the following super/subscripts: * indicates *p* <0.05 p-level significance, § indicates *p* <0.10 p-level significance. LSD post hoc analysis is indicated by the following superscripts: † represents *p* <0.05 difference from baseline value, Ψ represents *p* <0.05 difference from pre-run, ◊ represents *p* <0.05 difference from 60-min post, # represents *p* <0.05 difference from 24-hr post. *IL* interleukin, *RFT* race finish time, *G* group p-level, *T* time p-level, *G x T* interaction; *q* quadratic p-level, *L* linear p-level

### Clinical markers of immune-related complete blood counts

Table 9 demonstrates the immune response-related complete blood count marker results. All immune-related complete blood counts demonstrated significant changes over time (*p* <0.001), but no significant changes between groups.

**Table 9:** Markers of immune-related complete blood counts

Variable	Group	Baseline	Pre-Run	60-min Post	24-hr Post	48-hr Post	Group Mean	p-value (GG)	p-value (WSC)	RFT Covariate *p*-value (WSC)
Lymphocytes (K/uL)	P	1.83 ± 0.58	2.50 ± 0.81	1.47 ± 0.54	2.04 ± 0.69	2.26 ± 0.35	2.02 ± 0.11	*G =* 0.732		*G =* 0.414
	TC	1.82 ± 0.53	2.63 ± 0.72	1.43 ± 0.25	1.75 ± 0.45	2.18 ± 0.46	1.96 ± 0.14	*T <*0.001*	*T*_*q*_ *=* 0.013*	*T*_*q*_ *=* 0.509
	Time Mean	1.83 ± 0.11	2.56 ± 0.15^†^	1.45 ± 0.09^†Ψ^	1.90 ± 0.12^Ψ◊^	2.22 ± 0.08^†Ψ◊#^		*G X T =* 0.404	*G X T*_*L*_ *=* 0.905	*G X T*_*L*_ *=* 0.200
WBC (K/uL)	P	5.93 ± 1.45	6.54 ± 1.71	12.61 ± 3.39^^†Ψ^	6.64 ± 1.84^◊^	5.14 ± 0.84	7.37 ± 0.29	*G =* 0.314		*G =* 0.398
	TC	5.83 ± 1.50	6.85 ± 1.61	10.80 ± 3.40^†Ψ^	5.73 ± 1.20^◊^	5.33 ± 1.05	6.91 ± 0.35	*T <*0.001*	*T*_*q*_ *<*0.001*	*T*_*L*_ *=* 0.613
	Time Mean	5.88 ± 0.29	6.70 ± 0.33^†^	11.71 ± 0.66^†Ψ^	6.18 ± 0.32^◊^	5.24 ± 0.18^†Ψ◊#^		*G X T =* 0.223	*G X T*_*q*_ *=* 0.162	*G X T*_*q*_ *=* 0.500
MID (K/uL)	P	0.45 ± 0.14	0.53 ± 0.20	0.67 ± 0.23^†^	0.49 ± 0.19^◊^	1.10 ± 0.25^†Ψ◊#^	0.65 ± 0.03	*G =* 0.477		*G =* 0.607
	TC	0.43 ± 0.11	0.61 ± 0.19^†^	0.54 ± 0.15	0.40 ± 0.10^Ψ^	1.10 ± 0.32^†Ψ◊#^	0.61 ± 0.04	*T <*0.001*	*T*_*L*_ *=* 0.022*	*T*_*q*_ *=* 0.495
	Time Mean	0.44 ± 0.03	0.57 ± 0.04^†^	0.60 ± 0.04^†^	0.44 ± 0.03^Ψ◊^	1.10 ± 0.06^†Ψ◊#^		*G X T =* 0.276	*G X T*_*q*_ *=* 0.388	*G X T*_*q*_ *=* 0.562
GRAN (K/uL)	P	3.59 ± 1.07	3.53 ± 1.04	10.49 ± 3.49^^†Ψ^	4.09 ± 1.28^◊^	1.76 ± 0.59^†Ψ◊#^	4.69 ± 0.24	*G =* 0.349		*G =* 0.566
	TC	3.59 ± 1.41	3.64 ± 0.86	8.83 ± 3.35^†Ψ^	3.58 ± 0.98^◊^	2.05 ± 1.00^◊^	4.34 ± 0.29	*T <* 0.001*	*T*_*q*_ *<*0.001*	*T*_*L*_ *=* 0.326
	Time Mean	3.59 ± 0.24	3.58 ± 0.19	9.66 ± 0.67^†Ψ^	3.84 ± 0.23^◊^	1.90 ± 0.15^†Ψ◊#^		*G X T =* 0.259	*G X T*_*q*_ *=* 0.168	*G X T*_*q*_ *=* 0.493
GM-CSF (pg/mL)	P	26.38 ± 41.92	25.49 ± 31.29	26.08 ± 34.72	22.64 ± 30.21	22.41 ± 29.72	24.60 ± 9.15	*G =* 0.485		*G =* 0.696
	TC	42.26 ± 47.28	37.37 ± 45.40	35.27 ± 46.54	29.52 ± 38.02	29.41 ± 36.03	34.77 ± 11.04	*T =* 0.056^§^	*T*_*L*_ *=* 0.022*	*T*_*q*_ *=* 0.794
	Time Mean	34.32 ± 8.64	31.43 ± 7.36	30.68 ± 7.81	26.08 ± 6.57^†Ψ◊^	25.91 ± 6.34^†Ψ◊^		*G X T =* 0.407	*G X T*_*L*_ *=* 0.221	*G X T*_*L*_ *=* 0.319

Individual group and time data expressed as means ± SD, while group effects are presented as means ± SEM. Data represents the complete blood count immune response markers at each testing session during the 10 day intervention. MANOVA analysis revealed overall Wilks’ Lambda time (*p* <0.001) and group x time (*p* = 0.684). Univariate ANOVA p-levels from MANOVA analysis are presented for each variable. Univariate ANOVA p-levels are listed first by the Greenhouse-Geisser (GG) analysis and then by the within-subjects contrasts (WSC) to demonstrate the potential shape of the time or group x time interaction with significance indicated by the following super/subscripts: * indicates *p* <0.05 p-level significance, § indicates *p* <0.10 p-level significance. LSD post hoc analysis is indicated by the following superscripts: ^ represents *p* <0.05 difference between groups, † represents *p* <0.05 difference from baseline value, Ψ represents *p* <0.05 difference from pre-run, ◊ represents *p* <0.05 difference from 60-min post, # represents *p* <0.05 difference from 24-hr post. *WBC* white blood cell, *MID* mid-range absolute count, *GRAN* granulocyte absolute count, *GM-CSF* granulocyte-macrophage colony-stimulating factor, *RFT* race finish time, *G* group p-level, *T* time p-level, *G x T* interaction, *q* quadratic p-level, *L* linear p-level

### Muscle soreness perception assessment

**Fig. 7:**
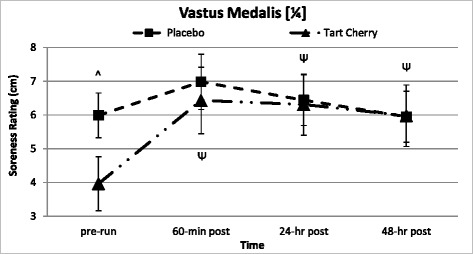
Perceptions of muscle soreness. Data expressed as means ± SE and significance indicated by the following super/subscripts: * indicates *p* <0.05 p-level significance, § indicates *p* <0.10 p-level significance. LSD post hoc analysis is indicated by the following superscripts: ^ represents *p* <0.05 difference between groups, Ψ represents *p* <0.05 difference from pre-run, ◊ represents *p* <0.05 difference from 60-min post, # represents *p* <0.05 difference from 24-hr post

Table 10 presents perceptions of muscle soreness. Perceptions were not measured at baseline. All locations of muscle soreness measurement demonstrated significant changes over time (*p* <0.001), peaking 60-min post-run. Significant differences between groups over time were found in vastus medalis (¼) soreness perception (*p* = 0.035) that was confirmed when accounting for running intensity discrepancies. Subsequent post-hoc analysis indicated significantly attenuated (34 %) pre-run vastus medalis (¼) soreness in TC compared to P with no differences in soreness perception between groups over the recovery (see Fig. 7). The change from pre-run vastus medalis (¼) soreness was smaller in P soreness perception compared to TC (*p* = 0.035) over the 48-h recovery. The other two locations of quadriceps soreness perception testing did not reveal any significant differences between supplementation groups.

**Table 10:** Quadriceps muscle soreness perception

Variable	Group	Pre-Run	60-min Post	24-hr Post	48-hr Post	Group Mean	*p*-value (GG)	*p*-value (WSC)	RFT Covariate *p*-value (WSC)
Algo I (cm)	P	5.99 ± 2.94^	6.98 ± 3.35	6.44 ± 2.69	5.95 ± 3.01	6.34 ± 0.66	*G =* 0.523		*G =* 0.660
	TC	3.96 ± 2.15	6.43 ± 3.15^Ψ^	6.31 ± 3.43^Ψ^	5.98 ± 3.04^Ψ^	5.67 ± 0.80	*T =* 0.003*	*T*_*q*_ *=* 0.002*	*T*_*L*_ *=* 0.440
	Time Mean	4.98 ± 0.52	6.71 ± 0.64^Ψ^	6.38 ± 0.59^Ψ^	5.96 ± 0.59^Ψ^		*G X T =* 0.110	*G X T*_*L*_ *=* 0.035*	*G X T*_*L*_ *=* 0.028*
Algo II (cm)	P	5.02 ± 2.55	5.77 ± 2.91	5.58 ± 2.56	4.78 ± 3.02	5.29 ± 0.60	*G =* 0.393		*G =* 0.847
	TC	4.15 ± 2.66	5.12 ± 3.01	4.51 ± 3.10	4.08 ± 2.30	4.47 ± 0.72	*T =* 0.122	*T*_*q*_ *=* 0.013*	*T*_*L*_ *=* 0.229
	Time Mean	4.59 ± 0.51	5.45 ± 0.58	5.05 ± 0.55	4.43 ± 0.54		*G X T =* 0.921	*G X T*_*q*_ *=* 0.889	*G X T*_*q*_ *=* 0.458
Algo III (cm)	P	4.65 ± 3.04^	6.41 ± 2.91^^Ψ^	6.04 ± 2.90^^Ψ◊^	4.41 ± 2.90^◊#^	5.38 ± 0.67	*G =* 0.098^§^		*G =* 0.226
	TC	2.59 ± 2.45	4.43 ± 2.89^Ψ^	3.48 ± 2.92	3.84 ± 3.11^Ψ^	3.58 ± 0.80	*T <* 0.001*	*T*_*q*_ *<* 0.001*	*T*_*L*_ *=* 0.508
	Time Mean	3.62 ± 0.55	5.42 ± 0.57^Ψ^	4.76 ± 0.57^Ψ^	4.12 ± 0.59^◊^		*G X T =* 0.058^§^	*G X T*_*q*_ *=* 0.053^§^	*G X T*_*q*_ *=* 0.257

Individual group and time data expressed as means ± SD, while group effects are presented as means ± SEM. Data represents the participant soreness perception in the quadriceps muscle group at each testing session during the 10 day intervention. MANOVA analysis revealed overall Wilks’ Lambda time (*p* <0.001) and group x time (*p* = 0.199). Univariate ANOVA p-levels from MANOVA analysis are presented for each variable. Univariate ANOVA p-levels are listed first by the Greenhouse-Geisser (GG) analysis and then by the within-subjects contrasts (WSC) to demonstrate the potential shape of the time or group x time interaction with significance indicated by the following super/subscripts: * indicates *p* <0.05 p-level significance, § indicates *p* <0.10 p-level significance. LSD post hoc analysis is indicated by the following superscripts: ^ represents *p* <0.05 difference between groups, Ψ represents *p* <0.05 difference from pre-run, ◊ represents *p* <0.05 difference from 60-min post, # represents *p* <0.05 difference from 24-hr post. *Algo I* Algometer location #1: Vastus Medalis 1/4, *Algo II* Algometer location #2: Vastus Lateralis 1/4, *Algo III* Algometer location #3: Vastus Lateralis 1/2, *RFT* race finish time, *G* group p-level, *T* time p-level, *G x T* interaction, *q* quadratic p-level, *L* linear p-level

## Discussion

Previous research has investigated the efficacy of tart cherry supplementation surrounding bouts of endurance exercise, however, this is the first study to investigate the effect of Montmorency tart cherry skin powder on acute endurance performance recovery. It was hypothesized that supplementation with this novel powdered tart cherry skin supplement prior to a single bout of endurance exercise would attenuate markers of muscle damage, oxidative stress, inflammation, and perceptions of muscle soreness in facilitation of faster recovery. Tart cherry ingestion reduced post-run serum markers of muscle catabolism, secondary muscle damage, and physiological stress over the 48-h recovery period compared to the placebo. Decreased muscle catabolism and stress are indicative of the attenuated recovery inflammatory response reported with tart cherry supplementation versus placebo. Antioxidant activity in those who ingested tart cherry was greater than the placebo, particularly 24 and 48-h post-run. Despite increases in actual over projected race pace times in both groups, the tart cherry group demonstrated smaller pace differences compared to placebo. Medial quadriceps soreness in tart cherry supplementers was significantly lower pre-run compared to those ingesting the placebo. However, results indicated a smaller change from pre-run medial quadriceps soreness in placebo supplementers over the 48-h recovery period compared to the tart cherry group.

Examining subject endurance performance, the increase in actual versus projected race pace irrespective of group is likely attributed to the 10-h fast and blood draw on the day of the endurance exercise challenge that would have not been experienced prior to any other race. The apparent beneficial effect of tart cherry powder supplementation on endurance performance through a decrease in race completion time is consistent with some of the previously published findings. Nieman et al. conducted a study in young, healthy males with 2-wk of quercetin supplementation (1000 mg/day) versus placebo [32]. Following a 12-min treadmill running trial at a 15 % grade and self-selected speed, Nieman et al. reported a significantly greater pre-supplementation versus post-supplementation change in distance covered with quercetin supplementation versus placebo [32]. The polyphenol content of a fruit-derived supplement similar to tart cherry was proven beneficial after extended supplementation in a study conducted by Kang et al. on regular endurance exercisers [33]. Kang et al. demonstrated that 30-d supplementation of oligomerized lychee fruit extract significantly elevated both submaximal running time and anaerobic threshold compared to a vitamin C/E mixture and a placebo [33].

Attenuation of muscle catabolism and secondary markers of muscle damage following prolonged endurance exercise physiologically provides the body optimal conditions for quicker recovery in preparation for subsequent performance bouts. Studying trained endurance runners, Kratz et al. [34] analyzed hemodynamic clinical chemistry makers before, 4 and 24-h post-Boston marathon. The results of demonstrated significant increases in total CK, AST, ALT, total protein, uric acid, total bilirubin, BUN, and creatinine 4-h post-race and confirmed significant elevations in CK, BUN, creatinine, uric acid, ALT, and AST over pre-race values 24-h post-race [34]. Bell et al. [35], in an acute endurance study following a combination of cycling sprints and time trials, reported results similar to the current study, demonstrating no differences in the CK response between Montmorency tart cherry concentrate and placebo supplementation. Unlike the current study, Howatson et al. [28] following marathon running demonstrated a trend of lower post-run CK levels when supplementing with tart cherry juice compared to placebo up to 48-h of recovery. Despite conflicting evidence among previous endurance-based tart cherry research, the post-run collective attenuation of these markers in the current study demonstrates a beneficial effect of powdered tart cherry supplementation on indices of muscle catabolism.

Previous research in the literature seems to conclude that the inflammatory process is mediated by both pro-inflammatory cytokines [36] and neuroendocrinological factors [37]. However, it has also been demonstrated that as major players in the development of secondary muscle damage, neutrophils, may also amplify the release of inflammatory cytokines [38, 39]. Nieman et al. [40] supplemented trained cyclists with quercetin, quercetin-EGCG (epigallocatechin 3-gallate), or placebo soft chews for 24-d surrounding 3-d of consecutive bouts of 3-h submaximal cycling. Nieman et al. [40] reported a significant decreases in plasma concentrations IL-6 immediately post-exercise on the third exercise day in the quercetin-EGCG group compared to placebo [40]. Howatson et al. [28] reported significantly lower inflammation immediately post-marathon through analysis of IL-6 that coincided with quicker recovery of knee extensor maximal strength following the marathon in Montmorency tart cherry juice supplemented subjects compared to placebo. Coinciding with the reduced inflammatory findings of Nieman et al. [40] and Howatson et al. [28] following endurance challenges, the current half-marathon study also reported a post-run attenuation in IL-6 with powdered tart cherry supplementation versus placebo. Similar to the post-exercise reduction in anti-inflammatory response (IL-10) in the quercetin-EGCG group published by Nieman et al. [40], the powdered tart cherry group in the current study also demonstrated similar changes via an attenuated IL-13 response over the 48-h recovery compared to placebo.

Glucocorticoids, specifically cortisol, released due to activation of the stress response through muscle mechanical microtrauma and ROS production have demonstrated an immunosuppressive influence. Davison and Gleeson [41], in an investigation of moderately trained males during 2.5-h moderate intensity cycling compared the effects of a beverage containing a vitamin C supplement with and without carbohydrate before and during endurance exercise. This study by Davison and Gleeson [41] revealed a significant increase in plasma cortisol levels immediately and 1-h post-exercise in both the placebo and vitamin C only supplemented groups with no significant difference between these two groups at 1-h post-exercise [41]. The addition of carbohydrates (alone or with vitamin C) significantly lowered the cortisol response during the exercise recovery up to 1-h post-exercise [41]. This result potentially demonstrates lower physiological stress post-exercise due to a higher energy state. The results of the current study revealed a similar cortisol response 60-min and 24-h post-run as both fasted groups demonstrated an increase from pre-run values, but the placebo group response from pre-run was significantly greater than the tart cherry group. This cortisol response group difference 60-min post-run may be due to a combination of anti-inflammatory and antioxidant effects of tart cherry anthocyanins.

Due to the reduced cortisol response 60-min post-run following tart cherry supplementation in the current study, it is likely that this anthocyanin-rich supplementation may modulate endogenous cytokine secretion following stressful exercise challenges. An acute supplementation study providing moderately active subjects with 48 g of anthocyanin-rich black currant extract immediately surrounding a single bout of high-intensity rowing conducted by Lyall et al. [42] demonstrated a significant post-exercise attenuation of pro-inflammatory cytokine production from LPS-stimulated cells. Lyall et al. [42] postulated from subsequent in vitro experimentation that this reduced cytokine production may have resulted from anthocyanin-based inhibition of NF-κB-mediated mechanisms. In the current study, attenuated IL-6 and serum cortisol levels during post-run recovery in tart cherry supplementers compared to placebo, demonstrates a potential relationship between the perception of physiological stress, regulation of anti-inflammatory cytokines, and cortisol release through NF-κB-mediation.

The attenuation muscle catabolic indices in the current study may also be partially attributed to an improved post-run redox balance with tart cherry supplementation compared to placebo. The greater antioxidant bioavailability from functional foods, such as tart cherries, containing high levels of flavonoids and anthocyanins [43], has been hypothesized to beneficially support endogenous antioxidant systems following strenuous exercise and excessive ROS-production. Howatson et al. [44] analyzed plasma TAS following a full marathon in trained endurance runners, and found that TAS was significantly greater in the tart cherry supplemented group compared to control up to 48-h post-race [7, 28, 38]. Unlike the tart cherry group, TAS levels dropped below baseline 48-h following endurance exercise in the placebo group as they failed to maintain redox balance. This discrepancy between supplementation groups demonstrates possible tart cherry antioxidant effectiveness on excessive ROS production during bouts of endurance exercise [7]. Similar to the recovery findings of Howatson et al. [44], the current study revealed a linear increase in TAS activity culminating in a 48-h recovery TAS activity that was greater in the tart cherry group compared to placebo. This demonstrates a potential short-term antioxidant effect of powdered tart cherry consumption surrounding a single endurance challenge with better achievement of redox balance compared to placebo supplementation.

Additional redox research has reported changes in exercise-induced oxidative stress utilizing TBARS analyses to measure lipid hydroperoxidation decomposition products over time. Supplementing with a tart cherry juice blend or placebo for 8-d surrounding a marathon run, Howatson et al. [28] demonstrated significantly lower TBARS levels 48-h post-marathon in the tart cherry supplemented group versus placebo. In coordination with Howatson et al. [28], Pilaczynska-Szczesniak et al. [26] reported significantly attenuated serum TBARS levels at 1-min and 24-h post-2,000 m incremental rowing test following 4-wk of chokeberry supplementation in trained rowing athletes compared to those supplemented with a placebo. In two more recent studies within the same trained rowing athlete population used by Pilaczynska-Szczesniak et al. [26], Skarpanska-Stejnborn et al. [45, 46] reported no differences in post-2,000 m incremental rowing test TBARS levels following 4-5-wk of supplementation with either *Rhodiola rosea L.* extract or artichoke extract. Contradictory outcomes across several studies may be due to mode of exercise, training and nutrition status, and duration of supplementation. Further, evidence in the literature utilizing lipid peroxidation (TBARS assays) analysis has presented a potential lack of oxidative damage detection specificity in human studies that may also explain the variability in results between the current and previous studies [4, 7, 35, 47].

As a highly reactive oxide metabolite of nitric oxide, peroxynitrate-bound tyrosine residues forming nitrotyrosine (NT) was measured by Sureda et al. [48] following supplementation of vitamin C + vitamin E surrounding a half-marathon. Suerda et al. [48] reported a significant increase in NT immediately post-race and 3-h post-race in the placebo group compared to the vitamin C + vitamin E supplemented group, indicating that antioxidant supplementation may have a dampening effect on oxidation of nitrogen-containing compounds with endurance exercise. The current study, however, reported no differences in NT levels over the study protocol or between supplementation groups. The outcome inconsistency may also be attributable to the differences in the antioxidant supplement and bioavailability, thus exhibiting a potential mechanistic variability in whole fruit-derived versus extracted antioxidant supplements (e.g. vitamins C and E).

The effect of phytochemical or vitamin containing anti-inflammatory supplements on the perception of muscle soreness after an endurance exercise challenge is inconsistent within the literature. Close et al. [20] acutely supplemented subjects with either ascorbic acid or a placebo surrounding an eccentrically braked endurance trial, where physically active subjects ran downhill continuously for 30-min. Close et al. [20] reported no significant differences in VAS pain ratings nor pressure algometry between groups up to 14-d post-exercise on six lower extremity locomotion muscle groups. With no effect on post-aerobic exercise delayed onset muscle soreness (DOMS) in addition to previous evidence in the literature, Close et al. [20] suggested a dissociation between post-exercise ROS production and DOMS. Following a marathon running event and a bout of high intensity stochastic cycling using a similar 200 mm VAS protocol, Howatson et al. [28] and Bell et al. [35] respectively, reported no difference in DOMS ratings between Montmorency tart cherry juice supplementation and placebo up to 72-h post-exercise. Within the current study, no quadriceps soreness differences were detected between groups over the three recovery time points. Due to the significant difference in pre-run medial quadriceps soreness between supplementation groups, delta changes calculated from pre-run revealed greater recovery medial muscle soreness with tart cherry supplementation compared to placebo. Without a baseline measure of quadriceps soreness perception and a subject training load record surrounding data collection, it is difficult to rationalize the pre-run muscle soreness discrepancy. However, the variability in soreness perceptions across muscle groups within the current study compared to previous research may also be due to the disparity in measurement protocol, supplements, exercise modality, and/or subject pool training status. Measurement of muscle soreness perception in the present study utilizing both an algometer and a GRPS was implemented to help ameliorate the purely subjective nature of a VAS as the only measure of pain or soreness.

The strengths of the current study revolve around the cohort of soreness measures and hemodynamic markers that contribute a more comprehensive analysis to the existing body of published literature. Some of the more recent endurance-based tart cherry supplementation research studies have investigated phytonutrient effectiveness on a comprehensive panel of hemodynamic markers, which will allow for parallels to be drawn to the powdered tart cherry supplement used in the current study. The current study did not enter the study cohort into a previously established race competition, but rather created a half-marathon race exclusively for the study. The utilization of this supplement within a trained population demonstrated its effectiveness under normative training, diet, and performance conditions. Potential limitations and weakness of the current study should also be considered. The placebo-control matched design of this study was effective in equalizing study subject exposure to the conditions of the half-marathon irrespective of supplement group. However, compared to previous research implementing a cross-over design, some variability associated with subject pairing may have been possible. Differences in aerobic state of training beyond the study inclusion/exclusion criteria may also have been a source of variability in study cohort recruitment. Due to the large number of hemodynamic markers measured in this study, the five selected time points of blood draws over the course of the experimental period may have not fully represented the pharmacokinetic profile of each marker. The major overriding strength of the current study is that this is the first study to be conducted utilizing a powdered form of tart cherries rather than a juice or concentrate.

## Conclusions

The findings of the current study revealed that consumption of a Montmorency powdered tart cherry supplement 7-d before, the day of, and 2-d after completing an endurance running challenge, appears to be an effective dietary supplement that may help attenuate post-run markers of muscle catabolism and physiological stress in aerobically trained individuals. Attenuation of inflammatory markers over the 48-h recovery also demonstrates significant promise with powdered tart cherry supplementation. Coupled with the dampening of the inflammatory response, the powdered tart cherry subjects seemed to better maintain post-run redox balance compared to placebo supplemented subjects. The initial effectiveness on aerobic performance, serum markers of muscle catabolism, physiological stress, and inflammatory mechanisms coupled with a more stable post-run redox balance potentially indicates a reduction in secondary muscle damage as a result of powdered tart cherry supplementation. Despite inconclusive evidence surrounding the perceptions of medial quadriceps soreness, the primary findings of the current study demonstrate that powdered tart cherry supplementation in endurance-trained individuals provides similar benefits as previously studied tart cherry juices or concentrates following acute bouts of aerobic-based exercise.
